# Prevalence and determinants of depression, anxiety and stress among psychiatric nurses in Ghana: a cross-sectional study

**DOI:** 10.1186/s12912-022-00964-5

**Published:** 2022-07-05

**Authors:** Sampson Opoku Agyemang, Jerry Paul Ninnoni, Nancy Innocentia Ebu Enyan

**Affiliations:** 1 Department of Mental Health, School of Nursing and Midwifery, College of Health and Allied Sciences, University of Cape Coast, Cape Coast, Ghana; 2 Department of Adult Health, School of Nursing and Midwifery, College of Health and Allied Sciences, University of Cape Coast, Cape Coast, Ghana

**Keywords:** Anxiety, Depression, Ghana, Psychiatric nurses, Risk factors, Stress

## Abstract

**Background:**

The job demand and stress associated with the nursing profession expose nurses to an increased risk of psychiatric morbidities such as anxiety and depression. This study assessed the prevalence of depression, anxiety and stress among psychiatric nurses in Ghana.

**Methods:**

A cross-sectional survey was conducted in three psychiatric hospitals in Ghana between March 2020 and May 2021. Simple random sampling technique were used to select 311 psychiatric nurses. Beck’s Depression Inventory, Beck’s Anxiety Inventory and Perceived Stress Scale were used to assess depression, anxiety and stress, respectively. Data were analysed using SPSS version 23.0.

**Results:**

The results showed that 19.6% of psychiatric nurses experienced mild to severe depression, 27% mild to severe anxiety and 42% mild to high stress. Regression analysis showed that participants with a diploma qualification had higher odds of having moderate depression compared to those with a master’s degree. In terms of stress, participants with a diploma qualification were 29.6% less likely to have moderate stress compared to those with a master’s degree. Those with a bachelor’s degree were 7.1% less likely to have moderate stress compared to those with a master’s degree.

**Conclusion:**

Psychiatric nurses experience depression, anxiety and stress to varying degrees. Education level was identified as a determinant of depression, anxiety and stress. Therefore, preventive strategies should be designed to reduce the risk of these conditions.

## Background

Depression, anxiety and stress are among the commonly reported mental health disorders and significantly contribute to the global burden of disease [1]. It is estimated that globally, 4.4% of the world’s population suffers from depression and 3.6% from anxiety disorders [2]. Depression is a severe mental health disorder with symptoms including loss of interest in pleasurable activities, feelings of sadness, guilt, low self-esteem, sleep disturbance and difficulties in concentration [3]. Out of about 450 million people who suffer from mental health disorders globally, approximately 150 million suffer from depression [4]. The manner an individual’s body responds to a perceived threat is known as anxiety [5]. Symptoms of anxiety include; increased blood pressure, respiration rate, pulse rate, tension, sweating and chest pain. Stress is a reaction that starts when an individual external or internal demand exceeds resources mobilised by the individual [6]. It is an individual’s feelings when she anticipates that her needs are more than the resources available to her to fulfil those demands [7]. When individuals cannot manage the negative impact of stress, they exhibit symptoms of anxiety and depression [8].

The nursing profession especially psychiatric nursing can be demanding and exposes nurses to work-related stress, anxiety, and depression [9]. The high prevalence of anxiety, depression, stress and burnout at the workplace among nurses reflects the nature of the nursing profession [10–13]. The job demand and pressure associated with the nursing profession put nurses at increasing risk of experiencing psychiatric morbidity such as anxiety and depression that can affect all aspects of their personal, family and professional life [11, 13, 14]. Depression is linked to dissatisfaction with life among nurses working in various health care settings including mental health [15]. Therefore, nurses’ mental health and well-being is important to deliver quality care and improve productivity.

A study conducted in Hong Kong reported that 35.8, 37.3 and 41.1% of nurses suffered from depression, anxiety and stress, respectively [16]. In another study, it was identified that 17 and 20% of midwives suffered from depression and anxiety, respectively [17]. Similarly, depression, anxiety and stress were prevalent among nurses in Africa. Evidence from Ethiopia showed that 22.9, 19.2 and 28.2% of nurses suffered from depression, anxiety and stress, respectively [8]. The COVID-19 pandemic heightened the stress levels of health workers, especially nurses [18, 19]. Comparatively, nurses felt more stressed during the COVID-19 pandemic than before the pandemic [18]. Another study in Portugal reported that COVID-19 led to an increase in the prevalence of depression, anxiety and stress among nurses compared to the general population [20].

Several factors are reported in the literature as predictors of depression, anxiety and stress among nurses. These include; lack of job satisfaction such as high workload, long working hours, sleep disturbance, conflict with colleagues and clients, lack of support from superiors, years of employment and marital status [7, 12, 16]. However, psychiatric nurses may even be at higher risk of developing depression, anxiety and stress because of the nature of the clinical environment, which sometimes can be hostile and require the control and restraint of patients who are aggressive [21–23]. In addition, workplace violence is common among psychiatric nurses and correlates significantly with occupational stress [24]. In China, stress among psychiatric nurses was rated higher than in other categories of nurses [21]. A study conducted among psychiatric nurses in Greece found that depression and anxiety are highly prevalent among psychiatric nurses [23]. Another study conducted in Japan found stress among mental health nurses to be associated with depression [22].

Although psychiatric nurses are considered to be at higher risk of developing depression, anxiety and stress compared to other categories of the nursing field [23], few studies have investigated the mental health of psychiatric nurses, especially in Ghana. For instance, a study conducted at the Pantang Hospital in Ghana reported that the higher the age of the nurse, the higher the risk of developing depression, anxiety and stress [25]. However, this study is limited in scope and was conducted in a single hospital.

Therefore, this study is a nationwide study aimed to assess the prevalence estimate of depression, anxiety, and stress among psychiatric nurses. Specifically, the study sought to: determine the prevalence of depression, anxiety and stress and identify associated socio-demographic factors among nurses in Ghanaian psychiatric hospitals.

## Methods

### Study design and study area

The study was a cross-sectional study conducted in three public psychiatric hospitals: Accra Psychiatric Hospital, Ankaful Psychiatric Hospital, and Pantang Hospital between March 2020 and May 2021. Accra Psychiatric Hospital is located in Adabraka in the Greater Accra Region of Ghana. The hospital was built in 1904 and commissioned in 1906 with a bed capacity of 200 patients. Currently, the hospital has a bed capacity of 600 patients. It is the oldest and most populated psychiatric hospital in Ghana [26]. Ankaful Psychiatric Hospital was built outside the nation’s capital after constructing the Accra Psychiatric Hospital. It was established in 1965 with a bed capacity of 500, but currently, the hospital has a total bed capacity of 311 [27]. The Pantang Hospital is the largest psychiatric hospital commissioned in 1975. It is a 500-bed facility located close to the Pantang village, 1.6 km off the Accra-Aburi Road and 25 km from Accra. The hospital is used as a training centre for nursing and medical students all over the country and beyond. The hospital also operates a polyclinic, maternity, child welfare clinic and an eye clinic that serves the general population [28].

### Population and sampling procedure

The population for this study included nurses who work in the three public psychiatric hospitals in Ghana. The study population comprised 993 nurses working in three public psychiatric hospitals in Ghana. This involved 462 nurses from Accra Psychiatric Hospital, 210 nurses from Ankaful Psychiatric Hospital, and 321 from Pantang Hospital. A probability proportionate to the size and simple random sampling technique were used to select participants.

The sample size was determined by applying Miller and Brewer’s formula [29]. It states that at 95% confidence level; $\mathrm{n}=\frac{N}{\left(1+N\ (a)2\right)}$. Where, n- desired sample size, N- target population, a- level of statistical significance of 0.05, 1-is a constant.

Therefore, the sample size, $\mathrm{n}=\frac{993}{\left(1+993(0.05)2\right)}=285.139=285.$

The calculated sample size of 285 was increased by 10% to 314 to ensure that samples were not lost during data collection and cleaning and increased the statistical power [30].

The sample size for each hospital was determined from the calculated sample size of 314, proportionate to the population of each hospital.

The sample size for Accra Psychiatric Hospital (*N* = 462)

$$\frac{462}{\ 993}x\ 314=146$$

The sample size for Ankaful Psychiatric Hospital (*N* = 210)

$$\frac{210}{\ 993}x\ 314=66$$

Sample size for Pantang Hospital (*N* = 321)

$$\frac{321}{\ 993}x\ 314=102$$

In all, 146 nurses were recruited from the Accra Psychiatric hospital, 102 from the Pantang hospital and 66 from the Ankaful psychiatric hospital.

After the determination of the sample size for each hospital, a simple random sampling procedure was used to obtain the required number of respondents from each hospital. The sample frame of the population for each hospital was determined by getting a list of all professional nurses working in that hospital from the nursing administration. A random number generator was used to generate random numbers and registered the name in the sample frame corresponding to the numbers to constitute the sample for that particular hospital. This was continued until the required number was met.

### Data collection

Three research assistants with bachelor’s degree in mental health nursing were recruited and trained to assist in the data collection process. The training involved ensuring proper administration of the questionnaire and ethical procedures including measures to ensure respondent’s confidentiality and anonymity during data collection. The training lasted for 2 h. The data were collected by the first author and research assistants at the three hospitals. The respondents filled in the self-administered questionnaires and returned them within 1 week. Three hundred and eleven questionnaires were returned (99.0% response rate) and entered into the Statistical Package for Social Sciences for analysis.

### Data collection instrument

Data were collected using a self-administered questionnaire. The instrument comprised 58 items which were divided into four sections; the first section focused on the socio-demographic characteristics of the respondents (e.g., “Gender”, “Age”, “Marital status”, “Educational status”, “Name of the hospital”, “Number of years spent in the nursing profession “, “Department/Ward”, “Monthly income”, and “Religion”).

The second section focused on the prevalence of depression. Beck’s Depression Inventory (BDI) was used to assess depression. The BDI is a 21-item self-reporting scale on a 4-point scale: 0- (Never- do not apply to me), 1- (Sometimes- applied to me to some degree), 2- (Often- applied to me to a considerable degree), 3- (Almost always- applied to me very much). Examples of some of the items are “I am so sad and unhappy that I can’t stand it”, “I feel I am a complete failure as a person”, and “I blame myself for everything bad that happens”. It has a minimum score of 0 and a maximum score of 63. The score of 0–9 indicates (minimal depression), 10–16 (mild depression), 17–29 (moderate depression) and 30–63 (severe depression) [31]. A cut-off score of 9 indicates a normal depression level. In this study, Cronbach’s alpha reliability coefficient for BDI was 0.91, demonstrating good internal consistency.

Anxiety was assessed using Beck’s Anxiety Inventory (BAI) in the third section. It is a 21-item self-report scale that is used to evaluate anxiety symptoms among adults on a 4-point Likert scale, which ranges from 0- (Not at all), 1- (Mildly- but it didn’t bother me much), 2- (Moderately- it wasn’t pleasant at times), 3- (Severely- it bothered me a lot). Some example items are “Wobbliness in legs”, “Unable to relax”, “Fear of worst happening”, and “Hands trembling”. A score of 0–7 indicates (low anxiety), 8–15 (mild anxiety), 16–25 (moderate anxiety) and 26–63 (severe anxiety). A cut-off score of 7 indicates a normal anxiety level [32]. The Cronbach’s alpha reliability coefficient for BAI was 0.91, indicating good internal consistency.

The fourth section of the instrument determined the prevalence of stress using the Perceived Stress Scale-10 (PSS). PSS is a 10-item scale used to assess respondents’ perception of stressful experiences over the previous month. Items on the scale are rated on a 5-point Likert scale which ranges from 0- (Never), 1- (Almost Never), 2- (Sometimes), 3- (Fairly Often), and 4- (Very Often). Some example items are “Upset because of something that happened unexpectedly?”, “You could not cope with all the things that you had to do?” and “Things were going your way?”. The scores were calculated after reversing the positive item’s score. It has a minimum score of 0 and a maximum score of 40; a high score indicates greater stress. Six out of the ten items of the PSS-10 are considered negative (1,2,3,4,5,6), and the remaining four are positive (7,8,9,10). A cut-off score of 13 indicates normal stress levels. A score of 0–13 represent (low stress), 14–26 (moderate stress) and 27–40 (high perceived stress) [33]. The Cronbach’s alpha reliability coefficient for PSS in the current study was 0.74, demonstrating good internal consistency.

Although there is no information regarding the validation of BDI, BAI and PSS in Ghana [34], these scales have been widely used in the Ghanaian setting [35–40] and found useful and applicable [41]. A cross-cultural study that involved Ghana, indicated that there was no significant difference in Becks; Depression Inventory scores among different countries [42]. Pre-testing of the instrument was conducted using 40 respondents at public hospitals with Mental Health Units within the Cape Coast Metropolis and Komenda-Edina-Eguafo-Abirem Municipality in Ghana to improve the validity and reliability of the instrument.

### Data analysis

The data were checked, cleaned, entered and analysed using the Statistical Package for Social Sciences (SPSS) version 23.0. A 95% confidence interval and *p-* < 0.05 were considered significant for this study. Frequencies and percentages were used to analyse the prevalence estimate of depression, anxiety and stress. Multinomial logistic regression was used to predict the socio-demographic characteristics influencing depression (minimal, mild, moderate, severe), anxiety (low, moderate, severe) and stress (low, moderate, high).

## Results

### Demographic data of respondents

Table 1 shows that 60.1% of the respondents were female, and 69.1% were aged 25–34 years. More than half (59.5%) of the respondents were diploma holders. The results further revealed that 28.3% of the respondents had spent 4 to 6 years in the nursing profession, and 50.2% of them earned between 1500 to 2000 Ghana cedis (the equivalent of 300–400 US dollars) monthly.

**Table 1:** Distribution of demographic variables of respondents *N* = 311

Variable	Frequency	Percentage (%)
Gender		
Male	124	39.9
Female	187	60.1
Age		
18–24	8	2.6
25–34	215	69.1
35–44	86	27.7
45 and above	2	.6
Marital status		
Single	140	45.0
Married	168	54.0
Divorced/separated	2	.6
Widowed	1	.3
Educational status		
Diploma	185	59.5
Bachelor’s degree	121	38.9
Master’s degree	5	1.6
Name of Hospital		
Ankaful Psychiatric Hospital	66	21.2
Accra Psychiatric Hospital	145	46.6
Pantang Hospital	100	32.2
Years spent in the nursing profession		
Less than 1 year	4	1.3
1–3	54	17.4
4–6	88	28.3
7–9	84	27.0
10 and above	81	26.0
Department/ward		
Administration	7	2.3
OPD	49	15.8
Acute ward	167	53.7
Chronic ward	88	28.3
Monthly income		
Less than 1500 cedis	40	12.9
1500–2000 cedis	156	50.2
2100–2900 cedis	106	34.1
3000 cedis and above	9	2.9
Religion		
Christianity	282	90.7
Islam	27	8.7
Traditional religion	2	.6

### Prevalence of depression, anxiety and stress among nurses in public psychiatric hospitals in Ghana

Table 2 shows that 15.1% of the study respondents experienced mild depression whilst 1.9% experienced severe depression. Additionally, it was found that 19.0 and 5.7% of the respondents experienced mild and moderate anxiety, respectively. Furthermore, it was observed that 42.8% of the respondents experienced moderate stress.

**Table 2:** Prevalence of depression, anxiety and stress* N* = 311

Variable	No.	%
Depression		
Minimal depression (0–9)	250	80.4
Mild depression (10–16)	47	15.1
Moderate depression (17–29)	8	2.6
Severe depression (30–63)	6	1.9
Anxiety		
Low anxiety (0–7)	227	73.0
Mild anxiety (8–15	59	19.0
Moderate anxiety (16–25)	18	5.7
Severe anxiety (26–63)	7	2.3
Stress		
Low stress (0–13)	176	56.6
Moderate stress (14–26)	133	42.8
High perceived stress (27–40)	2	0.6

### Socio-demographic characteristics that predict depression, anxiety and stress among nurses in public psychiatric hospitals in Ghana

Table 3 presents which socio-demographic characteristics of respondents predict depression. The model comprised six predictors, namely; gender, age, department, education status, hospital and income. The criterion variable was the depression levels of nurses working in psychiatric hospitals. The model fitting information for the above-specified model was significant, *p =* .008. The goodness of fit indices revealed that the data fit the model, *p =* .719. The Nagelkekerke pseudo-R-square value was .540 indicating that the socio-demographic variables of the respondents accounted for 54% of the variances in depression levels of mental health nurses. The educational level of respondents was found to be significantly associated with depression. Particularly, diploma and bachelor’s degree holders were more likely than master’s holders to be minimally depressed relative to severely depressed. It was found that the department (unit/ward) of the respondents was associated with depression. Those working in the Administration and Out-Patient Department (OPD) were less likely than those in Chronic Wards to be minimally depressed comparable to severely depressed. The income of nurses was positively and significantly associated with levels of depression. Nurses with income less than 1500 cedis equivalent to 300 United States dollars were more likely than those taking 3000+ cedis equivalent to 600 United States dollars to experience less depression relative to severely depressed.

**Table 3:** Socio-demographic variables prediction of depression

Depression		B	Std. Error	Wald	Sig.	Exp(B)
minimal	Intercept	.405	.913	.197	.657	
	Diploma	3.643	1.083	11.322	.001	38.222
	Bachelor’s Degree	3.611	1.159	9.713	.002	37.000
	Administration	−3.490	1.309	7.115	.008	.030
	OPD	−2.032	1.134	3.212	.007	.131
	Acute Ward	7.955	38.718	.042	.037	2849.968
	Less than 1500 cedis	18.177	40.354	.000	.996	783.199
	1500–2000 cedis	1.490	1.176	1.607	.205	4.437
	2100–2900 cedis	1.812	1.279	2.009	.156	6.125
	Ankaful Psychiatric Hospital	−1.558	1.166	1.784	.182	.211
	Accra Psychiatric Hospital	−.777	1.163	.447	.504	.460
	18–24 years	15.084	666.813	.982	.001	355.253
	25–34 years	3.674	1.485	6.121	.013	39.400
	35–44 years	4.382	1.736	6.374	.012	80.000
	Male	−1.372	.845	2.632	.105	.254
Mild	Intercept	−17.313	.802	466.272	.000	.736
	Diploma	18.412	1.043	311.776	.000	991.935
	Bachelor’s Degree	18.566	.000	231.667	.000	1156.757
	Administration	−19.028	4284.067	.000	.096	.449
	OPD	−2.303	1.396	2.719	.099	.100
	Acute Ward	7.900	38.722	.042	.838	2696.508
	Less than 1500 cedis	34.703	4061.354	7.021	.009	117.000
	1500–2000 cedis	18.484	1.021	313.954	.000	714.344
	2100–2900 cedis	18.090	67.000	3.713	.000	107.516
	Ankaful Psychiatric Hospital	−1.386	1.384	1.003	.317	.250
	Accra Psychiatric Hospital	−.288	1.302	.049	.825	.750
	18–24 years	16.911	3210.424	2.796	.000	220.221
	25–34 years	17.040	3109.286	1.906	.000	251.227
	35–44 years	17.183	3109.286	1.911	.000	290.416
	Male	−1.168	.977	1.429	.232	.311
moderate	Intercept	− 19.393	1.225	250.716	.000	123.451
	Diploma	18.294	1.683	118.119	.000	881.197
	Bachelor’s Degree	18.699	.000	114.181	.000	132.296
	Administration	−10.299	.704	42.310	.000	.336
	OPD	−2.002	167.881	.207	.009	.135
	Acute Ward	17.204	106.706	.026	.008	296.237
	Less than 1500 cedis	18.218	18,971.329	.000	.999	816.658
	1500–2000 cedis	18.777	16,902.236	.000	.999	142.193
	2100–2900 cedis	1.891	17,528.482	.000	.999	6.625
	Ankaful Psychiatric Hospital	−1.528	.000	.987	.873	.217
	Accra Psychiatric Hospital	18.045	.000	.564	.443	686.257
	18–24 years	16.911	1414.522	2.990	.000	220.267
	25–34 years	5.625	236.233	1.981	.001	277.188
	35–44 years	18.857	23.080	9.023	.000	154.868
	Male	−.916	1.643	.311	.577	.400

^a^The reference category for criterion variable: severe

Reference groups for predictors: education status- *masters*; department- *chronic Ward*; Income-*3000 and above*; Hospital- *Pantang*; age- *45 and above*; gender-*female*

Table 4 presents the analysis of which socio-demographic variables of respondents predict anxiety. The model comprised six predictors, namely; gender, age, department, education status, hospital and income. The criterion variable was the anxiety levels of nurses working in psychiatric hospitals. The model fitting information for the above-specified model was significant, *p =* .026. The goodness of fit indices revealed that the data fit the model, *p =* .059. The Nagelkekerke pseudo-R-square value was .380 indicating that the socio-demographic variables of the respondents accounted for 38% of the variances in anxiety levels of mental health nurses. The findings suggest that a higher level of education was associated with lower anxiety levels. This is to say that nurses who have diplomas and degrees were more likely to be anxious than those with master’s degrees. Nurses working at the Ankaful Psychiatric Hospital and Accra Psychiatric Hospital appeared to have higher anxiety levels than Pantang Hospital. This study indicates that the respondents’ age was associated with anxiety. Younger nurses were more likely to suffer from severe anxiety than older nurses. Furthermore, the results also found that male nurses were more likely than female nurses to have low pressure relative to severe anxiety.

**Table 4:** Socio-demographic variables prediction of anxiety

Anxiety		B	Std. Error	Wald	Sig.	Exp(B)
low	Intercept	20.335	1.803	127.237	.000	67.906
	Diploma	−.137	1827.846	192.000	.909	.872
	Bachelor’s Degree	−15.556	1.503	107.152	.000	.757
	Administration	16.736	1.296	166.872	.000	185.329
	OPD	16.779	5856.834	.001	.998	193.958
	Acute Ward	16.762	3170.651	.003	.996	190.986
	Less than 1500 cedis	−16.524	2322.514	.006	.994	.663
	1500–2000 cedis	.041	3058.412	.002	.900	1.041
	2100–2900 cedis	.062	1.279	.002	.961	1.064
	Ankaful Psychiatric Hospital	−.014	.852	1. 987	.007	.514
	Accra Psychiatric Hospital	−17.748	.742	571.722	.000	.659
	18–24 years	−7.736	128.941	34.952	.004	.390
	25–34 years	−7.692	24.253	76.751	.010	.683
	35–44 years	−14.969	1.735	74.396	.000	.155
	Male	17.516	.656	712.585	.000	404.460
moderate	Intercept	18.949	1.414	179.531	.000	12.987
	Diploma	−1.848	182.846	111.000	.045	.158
	Bachelor’s Degree	−18.949	127.000	219.000	.007	.896
	Administration	18.693	59.000	318.087	.076	131.333
	OPD	16.658	5856.834	.000	.908	171.650
	Acute Ward	17.222	3170.651	.000	.996	301.208
	Less than 1500 cedis	−18.082	2322.514	.000	.994	.403
	1500–2000 cedis	−1.099	3058.412	.000	.980	.333
	2100–2900 cedis	−1.810	.000	.000	.342	.164
	Ankaful Psychiatric Hospital	−.507	.000	.000	.326	.602
	Accra Psychiatric Hospital	−18.654	.000	.000	.792	.921
	18–24 years	−9.681	128.936	19.940	.006	.243
	25–34 years	−11.084	24.215	10.647	.021	.536
	35–44 years	−19.400	65.907	9.954	.000	.756
	Male	17.516	7.090	25.781	.000	403.461

^a.^The reference category for the criterion variable is: severe anxiety

Reference groups for predictors: education status- *masters*; department- *chronic*; Income-*3000 and above*; Hospital- *Pantang*; age- *45 and above*; gender-*female*

Results from Table 5 present which respondents’ socio-demographic characteristics predict their level of stress. The model comprised six predictors, namely; gender, age, department, education status, hospital and income. The criterion variable was the stress levels of staff working in psychiatric hospitals. The model fitting information for the above-specified model was significant, *p =* .043. The goodness of fit indices revealed that the data fit the model, *p =* .107. The Nagelkekerke pseudo-R-square value was .610 indicating that the socio-demographic variables of the respondents accounted for 61% of the variances in stress levels of mental health nurses. There appeared to be a negative association between educational level and stress. Nurses with higher academic levels were more likely to experience lower stress levels and vice versa. Additionally, it was found that nurses in the Administration and OPD were more likely to be highly stressed than those in the Acute and Chronic Ward. However, those in the Chronic Ward were more likely to have higher stress levels than those in the Acute Ward. It was observed that the income of nurses was negatively and significantly associated with stress levels. Thus, nurses who take a higher income were likely to experience low-stress levels than those with relatively more minor income. The result generally indicates that older nurses were likely to experience low stress levels and younger nurses were more likely to have high stress levels.

**Table 5:** Socio-demographic variables prediction of stress

Stress		B	Std. Error	Wald	Sig.	Exp(B)
low	Intercept	16.931	1.506	126.357	.000	24.12
	Diploma	−12.267	1.811	45.907	.000	.704
	Bachelor’s Degree	−12.741	1.133	126.457	.000	.929
	Administration	−.251	6810.055	98.000	.000	.285
	OPD	−16.382	1101.237	64.000	.008	.683
	Acute Ward	.009	.266	87.001	.042	1.991
	Less than 1500 cedis	−.096	1531.205	1.000	.000	.909
	1500–2000 cedis	−13.668	775.087	.986	.000	.156
	2100–2900 cedis	−.052	.699	.941	.005	.053
	Ankaful Psychiatric Hospital	−16.113	1124.354	.989	.000	.106
	Accra Psychiatric Hospital	−.139	.264	.599	.027	.870
	18–24 years	−.219	1132.185	1.268	.000	.245
	25–34 years	−11.248	326.098	.972	.001	.303
	35–44 years	−.080	1.431	9.955	.003	.083
	Male	−16.745	.239	4920.261	.000	.342
moderate	Intercept	15.544	1.009	237.230	.000	7.983
	Diploma	−11.188	1.425	61.615	.000	.385
	Bachelor’s Degree	−11.555	2.000	78.109	.000	.586
	Administration	−.391	810.550	.000	.900	.676
	OPD	−16.407	101.732	.000	.908	.488
	Acute Ward	.012	23.000	.000	.075	1.012
	Less than 1500 cedis	−.127	1531.205	1.0860	.000	.136
	1500–2000 cedis	−13.759	775.087	.986	.000	.056
	2100–2900 cedis	−.068	665.000	1.765	.000	.934
	Ankaful Psychiatric Hospital	−16.045	3452..456	.989	.000	.076
	Accra Psychiatric Hospital	.184	.000	.00	.064	1.202
	18–24 years	−.292	1132.185	1.000	.000	.747
	25–34 years	−11.561	326.095	.972	.001	.534
	35–44 years	−.107	98.090	2.456	.000	.899
	Male	−17.221	.604	238.054	.000	.318

^a.^The reference category for the criterion variable is: high stress

Reference groups for predictors: education status- *masters*; department- *chronic*; Income-*3000 and above*; Hospital- *Pantang*; age- *45 and above*; gender-*female*

## Discussion

Our study assessed the prevalence of depression, anxiety and stress among psychiatric nurses. We further identified the socio-demographic characteristics that predict depression, anxiety and stress among psychiatric nurses in Ghana. Our study revealed that most psychiatric nurses in Ghana experienced minimal depression and low anxiety. However, a substantial number of them experienced mild to moderate depression. We found that stress had a different trend of results. It was observed that although some psychiatric nurses experienced a low level of stress, a relatively high number of them were moderately stressed. Generally, it was evident that psychiatric nurses in Ghana experienced low to moderate stress levels. In this study, minimal depression and low anxiety do not necessarily mean that these two psychological distress variables are non-existent in the psychiatric nursing profession.

Contrary to these findings, other studies recorded moderate to high prevalence of depression, anxiety and stress among nurses [20, 24, 43, 44]. A study in Greece among psychiatric nurses concluded that 52.7 and 48.2% of psychiatric nurses were at risk of developing depression and anxiety, respectively [23]. Another study in China indicated high levels of stress among psychiatric nurses [21]. During the COVID-19 pandemic, psychiatric nurses in Germany experienced a high-stress level [18]. However, more than half of the nurses experienced low to moderate stress levels in an earlier study [44] which corroborates our research findings. These discrepancies observed between the results of this study and previous studies could be attributed to differences in the study settings and population. Whereas this study focused on psychiatric nurses in Ghana, previous studies have concentrated on general nurses’ psychological distress, with only a few focusing on psychiatric nurses [21, 23, 43].

Furthermore, there was a positive association between educational level and depression. Psychiatric nurses with bachelor’s and master’s degree were more likely to experience a high level of depression compared with those with a diploma. A study in public psychiatric hospitals in Greece found that psychiatric nurses with post-graduate degree were more likely to experience higher levels of depression compared to those with lower degrees [24]. Another study also found that nurses who obtained a university degree were more likely to develop depression and anxiety and found a positive association between higher education levels and stress [23]. Nurses with high educational levels often have high expectations of the profession and may be more involved in decision-making processes leading to anxiety and depression [23, 24]. The findings also suggest psychiatric nurses with a diploma qualification were more likely to experience higher anxiety and stress compared to those with a master’s degree. These findings are consistent with a study conducted in Taiwan, which found that nurses without degrees showed higher job stress levels than nurses with degrees [45].

The unit/ward where nurses work was significantly associated with depression and stress in our findings. We observed that psychiatric nurses working in the OPD were more likely to have higher stress and depression levels than those in the Acute and Chronic Wards. The OPD in psychiatric hospitals in Ghana comprises reception, emergency and assessment units. It serves as the first point of call for psychiatric emergencies, new clients and old clients who visit the hospital. Also, patients are presented at the OPD of a psychiatric hospital in a more distressed or agitated state and are more challenging to manage. Perhaps the increase workload at the OPD may have contributed to the high stress and depression levels.

The findings of this study reflect the views of scholars that stress has a significant relation with the unit/ward where nurses work. Nurses who work in units such as the emergency unit and outpatient department experience a high rate of stress than other nursing staff because of increased workload and dealing with crisis situations [9, 46].

It was also evident in the findings that the income of nurses was negatively associated with stress levels. Psychiatric nurses with higher incomes were less likely to experience stress. This implies that psychiatric nurses with low income experienced higher levels of stress. This finding is consistent with a previous study [7], which reported that middle to high-income earners was less likely to suffer from stress.

Furthermore, although gender was not significantly associated with depression, female nurses were found to be more likely to experience higher anxiety levels than male psychiatric nurses while male psychiatric nurses were more likely to experience higher stress levels than females. This finding is corroborated by [11] study, where male nurses had a significantly lower risk for anxiety when compared to female nurses.

### Strengths and limitations

Significant strengths of this study are that the sample size was large and representative of the population of psychiatric nurses in three public psychiatric hospitals in Ghana. Also, the structured questionnaire allowed the nurses to independently respond to the items on the questionnaire, which ensured increased reliability and validity. Our study used three different standardised psychometric scales to assess the prevalence of depression, anxiety and stress, which adds to the validity of the findings. However, this study did not include a comparison to conclude regarding depression, anxiety and stress levels in psychiatric nurses. The study was quantitative and cross-sectional in nature and was unable to explore participants’ experiences. A mixed methodology that includes some qualitative data may also have an added perspective to the study findings.

### Implications and future research

The study’s findings suggested that psychiatric nurses experience depression and anxiety and moderate stress level. In addition, the results showed that a higher level of education was associated with lower levels of anxiety and stress among psychiatric nurses. This highlights the need for hospital management to encourage further education and provide Continuing Professional Development (CPD) programmes on the management of depression, anxiety and stress by nurses to enhance patient care and improve their quality of life. Further education will have added value which may lead to improved salaries or income. The findings further indicate that psychiatric nurses who worked at the OPD were more likely to experience a high level of depression and stress than those in the Acute and Chronic wards. Therefore, stakeholders in mental health such as the Mental Health Authority need to design preventive strategies to reduce the risk of depression and stress at the OPD.

## Conclusion

A significant minority of psychiatric nurses in Ghana experience depression, anxiety and stress. Overall, the findings suggest the need for capacity building and resource provision in psychiatric hospitals across the country by the Ministry of Health and the Mental Health Authority. This will enhance nurses’ conditions of services leading to improved psychosocial wellbeing and better patient care. Psychiatric nurses who experience moderate to severe levels of depression, anxiety and stress should be provided with mental health care services such as counselling and psychosocial support.

## Data Availability

The datasets used and analysed during the current study are available from the corresponding author on reasonable request.

## Abbreviations

BAI: Beck’s Anxiety Inventory
BDI: Beck’s Depression Inventory
OPD: Out Patients Department
PSS: Perceived Stress Scale
SPSS: Statistical Package for Social Sciences
WHO: World Health Organization

## References

[1] Ferrari AJ, Somerville AJ, Baxter AJ, Norman R, Patten SB, Vos T (2013). Global variation in the prevalence and incidence of major depressive disorder: a systematic review of the epidemiological literature. Psychol Med.

[2] World Health Organization. Depression and other common mental disorders: global health estimates. Geneva World Health Organization. 2017;1(24).

[3] Marcus M, Taghi YM, van Ommeren M, Chisholm D, Saxena S. Depression: a global public health concern. World Mental Health Day. 2012.

[4] Murray CJL, Vos T, Lozano R, Naghavi M, Flaxman AD, Michaud C (2012). Disability-adjusted life years (DALYs) for 291 diseases and injuries in 21 regions, 1990-2010: a systematic analysis for the global burden of disease study 2010. Lancet..

[5] Clark D, Beck AT. Anxiety: A common but multifaceted condition. Cogn Ther Anxiety Disord Sci Pract. 2010:3–30 Available from: https://www.guilford.com/excerpts/clark5.pdf.

[6] Usman A, Akbar Z, Ahmed Z, Ahmed I (2011). Work stress experienced by the teaching staff of university of the Punjab, Pakistan: antecedents and consequences. Int J Bus Soc Sci.

[7] Yeshaw Y, Mossie A (2017). Depression, anxiety, stress, and their associated factors among Jimma university staff, Jimma, Southwest Ethiopia, 2016: a cross-sectional study. Neuropsychiatr Dis Treat.

[8] Clark DA, Beck AT (2010). Cognitive theory and therapy of anxiety and depression: convergence with neurobiological findings. Trends Cogn Sci.

[9] Khodadadi E, Hosseinzadeh M, Azimzadeh R, Fooladi M (2016). The relation of depression, anxiety and stress with personal characteristics of nurses in hospitals of Tabriz, Iran. Int J Med Res Heal Sci.

[10] Fradelos E, Tzitzikos G, Giannouli V, Argyrou P, Vassilopoulou C, Theofilou P (2014). Assessment of burn-out and quality of life in nursing professionals: the contribution of perceived social support. Health Psychol Res.

[11] Id CLH, Wu M, Ho C, Wang J (2018). Risks of treated anxiety, depression, and insomnia among nurses: a nationwide longitudinal cohort study. PLoS One.

[12] Koinis A, Giannou V, Drantaki V, Angelaina S, Stratou E, Saridi M. The impact of healthcare workers job environment on their mental-emotional health. Coping strategies: the case of a local general hospital. Health Psychol Res. 2015;3(1). 10.4081/hpr.2015.1984 PMC4768542 26973958

[13] Perry L, Lamont S, Brunero S, Gallagher R, Duffield C (2015). The mental health of nurses in acute teaching hospital settings: a cross-sectional survey. BMC Nurs.

[14] Papathanasiou IV (2015). Work-related mental consequences: implications of burnout on mental health status among health care providers. Acta Inform Med.

[15] Ghazwin MY, Kavian M, Ahmadloo M, Jarchi A, Javadi SG, Latifi S (2016). The association between life satisfaction and the extent of depression, anxiety and stress among Iranian nurses: a multicenter survey. Iran J Psychiatry.

[16] Cheung T, Yip PSF (2015). Depression, anxiety and symptoms of stress among Hong Kong nurses: a cross-sectional study. Int J Environ Res Public Health.

[17] Creedy DK, Sidebotham M, Gamble J, Pallant J, Fenwick J (2017). Prevalence of burnout, depression, anxiety and stress in Australian midwives: a cross-sectional survey. BMC Pregnancy Childbirth.

[18] Bartzik M, Aust F, Peifer C (2021). Negative effects of the COVID-19 pandemic on nurses can be buffered by a sense of humor and appreciation. BMC Nurs.

[19] Sampaio F, Sequeira C, Teixeira L (2021). Impact of COVID-19 outbreak on nurses’ mental health: A prospective cohort study. Environ Res.

[20] Sampaio F, Sequeira C, Teixeira L (2020). Nurses’ mental health during the Covid-19 outbreak: a cross-sectional study. J Occup Environ Med.

[21] Yao X, Shao J, Wang L, Zhang J, Zhang C, Lin Y (2021). Does workplace violence, empathy, and communication influence occupational stress among mental health nurses?. Int J Ment Health Nurs.

[22] Yoshizawa K, Sugawara N, Yasui-Furukori N, Danjo K, Furukori H, Sato Y (2016). Relationship between occupational stress and depression among psychiatric nurses in Japan. Arch Environ Occup Health.

[23] Tsaras K, Daglas A, Mitsi D, Papathanasiou IV, Tzavella F, Zyga S, Fradelos EC. A cross-sectional study for the impact of coping strategies on mental health disorders among psychiatric nurses. Health Psychol Res. 2018;6(1). 10.4081/hpr.2018.7466 PMC6247012 30596156

[24] Tsaras K, Papathanasiou IV, Vus V, Panagiotopoulou A, Katsou MA, Kelesi M, Fradelos EC. Predicting factors of depression and anxiety in mental health nurses: a quantitative cross-sectional study. Med Arch. 2018;72(1):62. 10.5455/medarh.2017.72.62-67 PMC5789556 29416221

[25] Atindanbila S, Abasimi E, Anim MT (2012). A study of work-related depression, anxiety and stress of nurses at Pantang hospital in Ghana. Res Humanit Soc Sci.

[26] Accra Psychiatric Hospital. Annual report 2019. Accra. 2019.

[27] Ankaful Psychiatric Hospital. Annual report 2019. Cape Coast. 2019.

[28] Pantang Hospital. Annual report 2019. Accra. 2019.

[29] Miller RL, Brewer JD (2003). The A-Z of social research- a dictionary of key social science research concepts.

[30] Bryman A. Why do researchers integrate/combine/mesh/blend/mix/merge/fuse quantitative and qualitative research. Adv Mixed Methods Res. 2008;21(8):87–100.

[31] Beck AT, Ward CH, Mendelson M, Mock J, Erbaugh J (1961). An inventory for measuring depression. Arch Gen Psychiatry.

[32] Beck AT, Epstein N, Brown G, Steer RA (1988). An inventory for measuring clinical anxiety: psychometric properties. J Consult Clin Psychol.

[33] Cohen S, Kamarck T, Mermelstein R. A global measure of perceived stress. J Health Soc Behav. 1983:385–96. 6668417

[34] Oppong S (2017). Contextualizing psychological testing in Ghana. Psychol a její kontexty.

[35] Stubbs B, Vancampfort D, Veronese N, Schofield P, Lin P-Y, Tseng P-T (2018). Multimorbidity and perceived stress: a population-based cross-sectional study among older adults across six low- and middle-income countries. Maturitas.

[36] Opoku-Acheampong A, Kretchy IA, Acheampong F, Afrane BA, Ashong S, Tamakloe B (2017). Perceived stress and quality of life of pharmacy students in University of Ghana. BMC Res Notes.

[37] Adzika VA, Glozah FN, Ayim-Aboagye D, Ahorlu CSK (2017). Socio-demographic characteristics and psychosocial consequences of sickle cell disease: the case of patients in a public hospital in Ghana. J Health Popul Nutr.

[38] Radcliffe C, Sam A, Matos Q, Antwi S, Amissah K, Alhassan A (2020). Sankofa pediatric HIV disclosure intervention did not worsen depression scores in children living with HIV and their caregivers in Ghana. BMC Public Health.

[39] Naab F, Brown R, Heidrich S (2013). Psychosocial health of infertile Ghanaian women and their infertility beliefs. J Nurs Scholarsh.

[40] Nkyi AK, Ninnoni JPK (2020). Depression, purpose in life, loneliness and anxiety among patients with substance use disorders in Ankaful psychiatric Hospital in Ghana. J Psychosoc Rehabil Ment Heal.

[41] Tashakkori A, Barefoot J, Mehryar AH (1989). What does the beck depression inventory measure in college students?: evidence from a non-western culture. J Clin Psychol.

[42] Dorahy MJ, Lewis CA, Schumaker JF, Akuamoah-Boateng R, Duze MC, Sibiya TE (2000). Depression and life satisfaction among Australian, Ghanaian, Nigerian, northern Irish and Swazi University students. J Soc Behav Pers.

[43] Jordan TR, Khubchandani J, Wiblishauser M (2016). The impact of perceived stress and coping adequacy on the health of nurses: a pilot investigation. Nurs Res Pract.

[44] Maharaj S, Lees T, Lal S. Prevalence and risk factors of depression, anxiety, and stress in a cohort of Australian nurses. Int J Environ Res Public Health. 2019;16(1):61. 10.3390/ijerph16010061 PMC6339147 30591627

[45] Chen CH, Wang J, Yang CS, Fan JY (2016). Nurse practitioner job content and stress effects on anxiety and depressive symptoms, and self-perceived health status. J Nurs Manag.

[46] Davey A, Sharma P, Davey S, Shukla A (2019). Is work-associated stress converted into psychological distress among the staff nurses: a hospital-based study. J Fam Med Prim Care.

